# Physician Perceptions about the Barriers to Prompt Diagnosis of Mild Cognitive Impairment and Alzheimer's Disease

**DOI:** 10.1155/2019/3637954

**Published:** 2019-05-21

**Authors:** Davneet Judge, Jenna Roberts, Rezaul Khandker, Baishali Ambegaonkar, Christopher M. Black

**Affiliations:** ^1^Adelphi Real World, Grimshaw Lane, Cheshire SK10 5JB, UK; ^2^Merck & Co., Inc., 2000 Galloping Hill Road, Kenilworth, New Jersey 07033, USA

## Abstract

Prior studies have identified numerous barriers to the prompt diagnosis of patients with suspected Alzheimer's disease (AD). The aim of the study was to evaluate physician's perceptions of the importance of previously identified barriers to diagnosis, but with a specific focus on the presentation of mild cognitive impairment (MCI), which may be indicative of neurodegenerative disorders such as AD. A second aim was to evaluate how the perspective of primary care physicians (PCPs) may differ from that of specialists. A cross-sectional online survey of PCPs and specialists who routinely manage patients with complaints of age-related cognitive impairment was conducted. Participants were asked to identify barriers to prompt diagnosis from prespecified lists of known diagnostic challenges categorized into 4 domains: patient-related, physician-related, setting-related, and those relating to the clinical profile of AD. Physicians report a range of barriers when attempting to diagnose MCI and AD. Major themes included patients seeing cognitive decline as a normal part of aging and not disclosing symptoms, long waiting lists, and a lack of treatment options and definitive biomarker tests. Generally, PCPs and specialists showed broad agreement; however, PCPs were more likely to identify burdens on the healthcare system, such as long waiting lists and inadequate time to evaluate patients. Substantial barriers continue to hinder early diagnosis of MCI and AD. There are numerous areas where improvements might be made but the implementation of potential interventions will likely be associated with financial strain for many healthcare systems.

## 1. Introduction

Prompt diagnosis of Alzheimer's disease (AD) has been advocated with the belief that it may be associated with a number of benefits such as better coordination of care, the opportunity for patients and caregivers to plan for the future, and the postponement of institutionalization [1, 2]. In practice, this requires case detection during prodromal or predementia phases, which may precede AD by a number of years [1]. There has been growing recognition of the importance of this early disease stage when symptoms are limited to mild cognitive impairment (MCI). Indeed, recent and ongoing clinical trials in AD continue to experiment with very early drug interventions in attempts to develop a disease modifying agent but unfortunately, so far, these attempts have failed and there are no such approved agents currently available [2]. It is perhaps unsurprising that within the context of limited treatment options, drawbacks to early diagnosis have also been recognized, such as a potential stigma associated with the disease and increased risk of suicidal behavior in vulnerable patients [3]. However, the range of benefits that an early diagnosis can provide to patients, such as enabling access to available medication, counseling, ruling out other conditions, and allowing patients and their families to make legal and financial plans, are often thought to offset potential negative consequences [4]. Despite this, evidence suggests that missed or delayed diagnosis of dementia is substantial in primary care, and diagnostic delays, errors or uncertainty, and/or even a complete lack of formal diagnosis in some patients has been described [5–8].

Several authors have attempted to identify the challenges associated with achieving early and prompt diagnosis of dementia, AD, and MCI [3–7, 10, 11]. Koch an Iliffe [6], for example, reviewed 11 studies examining barriers to the diagnosis and the management of patients with dementia from the perspective of PCPs. Six common themes were identified including lack of support for patients, caregivers, and physicians, time and financial constraints, stigma, diagnostic uncertainty, and concerns around disclosure of the diagnosis. The authors concluded that there was a need for significant improvements in service provision and that improved communication between primary and secondary care would be particularly important. Overlapping challenges were identified in the 2009 review by Bradford et al. exploring missed and delayed dementia diagnoses [5], as well as the more recent 2016 review by Dubois et al. which focused specifically on MCI and AD [2]. Collectively, these authors highlight the need for system level changes with coordinated national strategies required to achieve this. Toward these ends, the World Health Organization has recognized the importance of timely diagnosis in its Global Action Plan on the Public Health Response to Dementia, [12] and in turn, a number of countries have enacted formal national strategies that include efforts to educate physicians about the importance of prompt diagnosis of AD and other dementias [13–16].

These efforts will be supported by a thorough understanding of the current factors that hinder this process. A detailed understanding of the challenges faced by clinicians can be used to inform interventions aimed at overcoming the issues and to guide resources and efforts toward specific areas where improvements are required. To date, most studies and reviews have examined the primary care setting [5, 6, 11], and it is unclear if similar barriers affect the diagnostic process in secondary care. When specialists have been consulted about these issues, it has generally been a small sample of experts whose views and experiences may not represent the broader specialist physician community. In addition, previous research has more commonly explored diagnostic challenges associated with dementia in general rather than AD, and there has also been far less focus on MCI as a precursor to AD. Although these are related concepts, it is conceivable that there are factors that are specific to each of these conditions.

To address this gap in the literature, and to obtain up-to-date information from the primary care setting, we conducted a large-scale survey across five European countries, Canada, and the USA. A broad range of physicians were recruited to participate including PCPs and a range of specialists responsible for treating cognitive impairment and AD. The aim of the study was to understand current clinical practices and barriers related to the diagnostic process for patients who present with suspected MCI or AD. The second aim of the study was to evaluate how the perspective of primary care physicians (PCPs) may differ from that of specialists.

## 2. Materials and Methods

This was a multicountry, cross-sectional physician survey conducted with primary care and secondary care physicians (geriatricians, neurologists, psychiatrists, and psychogeriatricians) from Europe (France, Germany, Italy, Spain, and the UK), the USA, and Canada. Physicians were recruited from a preexisting list of practitioners who had agreed to be contacted about participation in research. In order to be eligible for the study, and to ensure sufficient experience with treating and diagnosing AD, PCPs had to have seen at least 5 patients with MCI or AD over the past six months, and specialists had to have seen at least 10 such patients over the same time period. Quotas were set in an attempt to recruit a specific number of primary and secondary care physicians across the countries of interest. We targeted 140 specialists and 60 PCPs from each European country, 100 specialists and 75 PCPs from Canada, and 75 specialists and 150 PCPs from the USA. Quotas for subcategories of physician specialists were not preset. These targets were based on a consideration of population density as well as a pilot research, which indicated a more involved role in the AD diagnosis for PCPs in the USA relative to those practicing across Europe.

Data were collected in quarter 4 of 2017 in the form of an online survey. A predefined list of barriers was included based on a review of the published literature [2, 5–7, 11] and these were separated into four categories associated with the patient and family (e.g., “patients do not disclose symptoms”), healthcare setting (e.g., “too much strain on limited resources”), factors relating to physicians themselves (e.g., “I do not feel like I have adequate training to diagnose AD”), and factors relating to the clinical setting/clinical profile of AD (e.g., “lack of definitive biomarker tests”). One item in the healthcare setting category (“delay in referral from primary care”) was shown only to specialists. Physicians were asked to select which of these items they thought were the main challenges or barriers they faced when thinking about early/prompt diagnosis of MCI that could progress to AD. They were permitted to select as many as they felt were applicable. Alternatively, for each of the four categories (patients/ families, healthcare setting, physicians themselves, or the clinical setting/profile of AD) they could select that this was not a key issue. If this was chosen, all other items in that category became mutually exclusive.

### 2.1. Statistical Analyses

The study was designed to be descriptive only with no formal hypothesis testing. The reported statistics depended on the type of variable described: for numeric variables, the mean and standard deviation are reported; for categorical variables the number and percentage are shown. There was no missing data and logic checks contained within the online survey forced a response to each question. All analyses were performed with IBM® SPSS® Data Collection Survey Reporter Version 7.

## 3. Results

In total, 1365 physicians completed the survey and the target sample was met in all countries with the exception of Canada. The distribution of respondents according to the specialty and country in which they practice is shown in Table 1. Only 1% of the physician sample had qualified within the last four years. Overall, 27% had qualified within the last 5-15 years, 37% had qualified within the last 16-25 years, 31% has qualified within the last 26- 38 years, and 4% had qualified over 38 years ago.

On average, PCPs who participated in the survey had 62 patients with MCI and 43 patients with AD under their care during the preceding month. In contrast, specialists had 60 MCI patients and 88 AD patients under their care, on average, during the preceding month.

**Figure 1:**
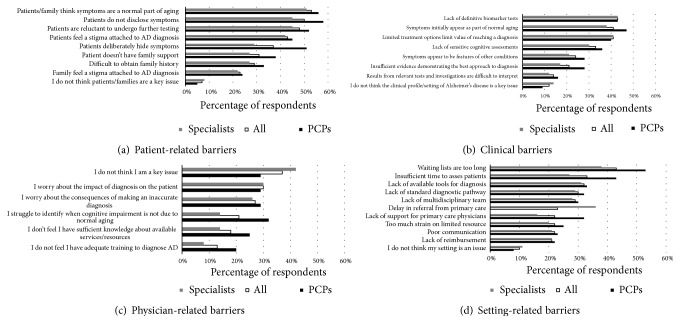
Barriers to prompt diagnosis of MCI or AD as perceived by physician respondents. *∗* Abbreviations: PCPs, primary care physicians; AD, Alzheimer's disease.

The frequencies at which physician identified specific barriers relating to the patient, physician, setting, and AD are shown in Figures 1(a)-1(d).

### 3.1. Patient-Related Barriers

The most commonly identified patient-related factors were patients and family thinking that symptoms were a normal part of aging (53%) and patients not disclosing symptoms (50%). Unwillingness of patients to undergo further testing was a common response (48%) and according to both PCPs (24%) and specialists (22%) patients and their families still felt that there was a stigma attached to a diagnosis of AD. In almost every instance, each of the prespecified patient-related barriers was identified by a higher percentage of PCPs than specialists but this was particularly the case for reports of patients deliberately hiding symptoms and patients not disclosing symptoms. At a country level, German physicians were more likely to indicate problems with patients hiding symptoms (65%) compared to other countries, and US physicians were more likely to indicate that a lack of family support for patients was an issue (42%). Canadian physicians commonly reported difficulties obtaining family history (41%).

### 3.2. Clinical Barriers

The lack of definitive biomarker tests was the most commonly identified clinical barrier overall in this category (43%), supporting the need for continued research in this arena. The response “limited treatment options limit the value of reaching a diagnosis” was also commonly identified (41%). Interestingly, another commonly identified barrier was that symptoms initially appear as part of normal aging (41%), perhaps validating the similar patient-related barrier. This was reported by specialists (38%) as well as PCPs (47%).

### 3.3. Physician-Related Barriers

Among the four categories of diagnostic barriers, respondents identified physician-related barriers the least frequently overall. Indeed, the most common response in this category selected by 42% of specialist and 29% of PCPs was “I do not think I am a key issue.” However, 37% of physicians overall stated that they worried about the impact of diagnosis on the patient. The most common response among PCPs was that they struggled to identify when cognitive impairment was not present due to normal aging (32% of respondents), which was consistent with barriers identified in the two previous domains of responses. Approximately 30% of both PCPs and specialists indicated that an important barrier was their concern about the impact of the diagnosis on the patient or the consequences of an inaccurate diagnosis. PCPs were more likely than specialists to indicate that they had insufficient knowledge about available services or resources (25% vs. 14%) or that they had inadequate training to diagnose AD (20% vs. 8%).

### 3.4. Setting-Related Barriers

The most commonly identified barrier related to their healthcare setting selected by 43% of respondents overall was that waiting lists were too long, and similarly insufficient time to assess patients was often identified (33%). These were particularly common complaints among PCPs (53% and 43%, respectively) compared to specialists (38% and 27%, respectively). Specialists also identified a delay in referral from primary care as a significant barrier (36%). Approximately 30% of both PCPs and specialists identified lack of available tools for diagnosis or lack of a standard diagnostic pathway as important barriers to a prompt diagnosis.

## 4. Discussion

Previous studies and reviews have enumerated many of the barriers that may be preventing prompt diagnosis of patients who present with evidence of cognitive decline [2, 5, 6, 11]. The goals of the present study were, first, to evaluate the current state of physician perceptions about such barriers given the initiation of organized efforts to improve the diagnostic process and, second, to extend existing knowledge by including the perceptions of specialist physicians with a specific focus on the diagnosis of patients with MCI or AD.

Our survey reaffirmed many of the barriers to prompt diagnosis described in prior studies and gave some measure of the relative importance of those barriers from the perspective of physicians. A common theme in prior studies is that patients and family members often believe that symptoms of cognitive decline are part of normal aging [2, 5, 6, 11]. This factor was the most commonly identified barrier overall in our study, too. It is also interesting that some physicians recognized a similar barrier affecting themselves: that they struggled to know when cognitive impairment was not due to normal aging (21% of respondents). In addition, 41% of respondents identified their perception that symptoms initially appeared as a part of normal aging as a barrier associated with the clinical profile of AD. This finding reaffirms the importance of continued research toward the development of more sensitive and specific tests, including cognitive tests and biomarker tests, that can distinguish the cognitive signs of normal aging from the cognitive declines associated with specific dementing illnesses such as AD. At first glance the call for better tests may conflict with the finding that many patients are reluctant to undergo further testing (as reported by 48% of physicians in this survey). However, a simple, economical, yet specific and sensitive test may obviate the need for many of the tests currently used, potentially reducing the overall burden of testing on patients while improving rates of prompt diagnosis [17]. Alternatively, it is conceivable that patients and their families are not concerned about the tests themselves, but rather it is the potential outcome of such tests (i.e., dementia diagnosis) that is driving this. If this is true, it will be important for physicians to demonstrate to patients the benefits of undergoing such tests in terms of future disease management.

Another common theme in our study and prior studies is the continued existence, among both patients and family members, of a stigma associated with cognitive decline, which may be amplified by a medical diagnosis of MCI or dementia. This barrier may also be related to several other identified barriers such as deliberate attempts by patients to hide symptoms or not to disclose symptoms, and concerns among physicians about how the diagnosis could impact the patient. Increased efforts with educational campaigns and support services will likely be required to address this.

An additional aim of the study was to identify the barriers from the perspective of specialist physicians as well as PCPs: in general, there were many points of agreement between the two sets of physicians. Specialists indicated that one of the barriers to prompt diagnosis was a delay in referral from primary care. However, in turn PCPs more commonly mentioned that patients deliberately hiding symptoms and not disclosing symptoms constitute an issue. It is therefore unclear whether a delay to reach secondary care is driven by PCP referral timelines per se or if this actually occurs because PCPs are often managing patients who delay discussing symptoms that would be indicative of MCI.

With regard to factors directly related to physician's themselves, PCPs were more likely than specialists to indicate that they lacked knowledge about available resources or that that they had inadequate knowledge to diagnose AD. These findings may be useful for identifying target audiences for interventions intended to improve diagnostic capability among PCPs. Another notable difference between PCPs and specialists was that PCPs were more likely to identify barriers related to strains on the healthcare system, such as the fact that waiting lists are too long and that they have insufficient time to assess patients. These observations join the large chorus of voices pointing out the global burden of disease, the increasing portion of that burden that is due to AD and other dementias, and shortages in the number of PCPs able to provide care for an aging population [18–20]. More focus is needed to ensure physicians have the awareness and available resources in place to action the existing recommendations [9] for how to best manage AD patients.

## 5. Limitations of the Study

This study identified only barriers to diagnosis as perceived by physicians. Thus, it should not be interpreted as an objective measure of the actual barriers but rather a subjective narrative collected from the physicians. The strength of our study lies in the large number of participating physicians covering multiple countries and specialties; hence we believe our findings contribute to the current knowledge in the field of AD, MCI, and early diagnostics. However, we acknowledge that many of the potential barriers included in this survey could be explored in significantly more depth. For example, the physician-related barrier “I worry about the impact of diagnosis on the patient” could relate to numerous different specific areas of concern. Qualitative research where these themes are explored in more detail might help reveal more about the underlying factors. The results from this survey may help direct this future research by initially highlighting the barriers that appear to be of most relevance to physicians.

Our sample included physicians across multiple specialties. Given that a multitude of physicians and other healthcare professionals deal with diagnosing and managing AD/MCI patients in the real world, we hope that the heterogeneous group of physicians participating in our study might reflect the clinical situation at large. However, unfortunately, no data about physicians' response rates were available. Therefore, the representativity of the sample needs to be considered when interpreting the results and drawing conclusions.

As a related point, it is likely that other stakeholders such as patients, family members, caregivers, and other healthcare providers (e.g., nurses) will have different perceptions about some of the barriers, or at least the relative importance of the various barriers. Involving this wider audience will be an important avenue for future research to gain a holistic overview of the challenges. Lastly, the respondents in our survey were presented with prespecified lists of potential barriers that had been identified from the published literature and pilot work. This structure provided limited opportunities for other barriers to be identified.

## 6. Conclusions

Prior studies have identified multiple barriers that may prevent prompt diagnosis of patients with suspected dementia. Our study reaffirms that many of those barriers continue to contribute to diagnostic delays and that challenges are faced at both the primary and secondary care level. There are numerous areas where improvements may be required including sensitive and specific diagnostic tests, increased physician and patient education, and reduced referral times. However, the implementation of any such changes will be subject to available funding and will likely place significant financial strain on healthcare budgets.

## Figures and Tables

**Table 1:** Distribution of survey respondents across countries and across medical specialties.

	n (%)						
	USA	Canada	France	Germany	Italy	Spain	UK
Total sample	225	140	200	200	200	200	200
*Primary care*	*150 (67)*	*50 (36)*	*60 (30)*	*60 (30)*	*60 (30)*	*60 (30)*	*60 (30)*
*Secondary care*	*75 (33)*	*90 (64)*	*140 (70)*	*140 (70)*	*140 (70)*	*140 (70)*	*140 (70)*
Geriatrician	-	17 (12)	30 (15)	11 (6)	22 (11)	10 (5)	35 (18)
Neurologist	75 (33)	26 (19)	64 (32)	81 (41)	73 (37)	91 (46)	25 (13)
Psychiatrist	-	37 (26)	39 (20)	43 (22)	43 (22)	36 (18)	37 (19)
Psychogeriatrician	-	10 (7)	7 (4)	5 (3)	2 (1)	3 (2)	43 (22)

*∗* Predefined quotas were set for the target number of specialists vs. primary care physicians across each country taking into account population density. The table is reproduced from a previous publication describing other aspects of the data [9].

## Data Availability

The survey data used to support the findings of this study are included within the article, and any supporting literature has been referenced within the reference list.
